# Effect of the Adaptive Response on the Wear Behavior of PVD and CVD Coated Cutting Tools during Machining with Built Up Edge Formation

**DOI:** 10.3390/nano10122489

**Published:** 2020-12-11

**Authors:** German Fox-Rabinovich, Iosif S. Gershman, Kenji Yamamoto, Julia Dosbaeva, Stephen Veldhuis

**Affiliations:** 1 Department of Mechanical Engineering, McMaster Manufacturing Research Institute (MMRI), McMaster University, Hamilton, ON L8S 4L8 Canada; jdosby@mcmaster.ca (J.D.); veldhu@mcmaster.ca (S.V.); 2 Joint Stock Company Railway Research Institute, Moscow State Technological University “Stankin” (MSTU “STANKIN”), 127994 Moscow, Russia; isgershman@gmail.com; 3 Applied Physics Research Laboratory, Kobe Steel Ltd., 1-5-5 Takatsuda-dai, Nishi-ku, Kobe, Hyogo 651-2271, Japan; yamamoto.kenji1@kobelco.com

**Keywords:** built-up edge formation, self-organization, self-organized criticality, the adaptive response of surface engineered system

## Abstract

The relationship between the wear process and the adaptive response of the coated cutting tool to external stimuli is demonstrated in this review paper. The goal of the featured case studies is to achieve control over the behavior of the tool/workpiece tribo-system, using an example of severe tribological conditions present under machining with intensive built-up edge (BUE) formation. The built-ups developed during the machining process are dynamic structures with a dual role. On one hand they exhibit protective functions but, on the other hand, the process of built-up edge formation is similar to an avalanche. Periodical growth and breakage of BUE eventually leads to tooltip failure and catastrophe of the entire tribo-system. The process of BUE formation is governed by the stick–slip phenomenon occurring at the chip/tool interface which is associated with the self-organized critical process (SOC). This process could be potentially brought under control through the engineered adaptive response of the tribo-system, with the goal of reducing the scale and frequency of the occurring avalanches (built-ups). A number of multiscale frictional processes could be used to achieve this task. Such processes are associated with the strongly non-equilibrium process of self-organization during friction (nano-scale tribo-films formation) as well as physical–chemical and mechanical processes that develop on a microscopic scale inside the coating layer and the carbide substrate. Various strategies for achieving control over wear behavior are presented in this paper using specific machining case studies of several hard-to-cut materials such as stainless steels, titanium alloy (TiAl6V4), compacted graphitic iron (CGI), each of which typically undergoes strong built-up edge formation. Various categories of hard coatings deposited by different physical vapor deposition (PVD) and chemical vapor deposition (CVD) methods are applied on cutting tools and the results of their tribological and wear performance studies are presented. Future research trends are outlined as well.

## 1. Introduction

Adaptive response to external stimuli is mostly observed and studied in natural systems [1]. This phenomenon is directed at protecting a system against damage [2]. In recent times, several scientific institutions have gained interest in conducting research on this effect in the field of nanomaterials and tribology [3,4]. Adaptive response in tribo-systems strives to limit the influence of external stimuli, i.e., friction. The major consequences of friction are wear and surface damage. An adaptive response can reduce the intensity of both processes, and as a result, lower the entropy production [5]. The processes associated with adaptive response develop at two different scales:

- The nano-scale process of tribo-film formation (a non-equilibrium process related to self-organization) caused by the indirect influence of friction [5]. This process is initiated by the surface modification of cutting tools with further interaction with the environment (tribo-oxidation), which forms protective/lubricating tribo-films [5,6]. Such processes commence abruptly and result in negative entropy production [6]. Although they are not directly related to friction, a part of friction energy is consumed during tribo-film generation, which would be otherwise spent on the wear process. As a result, the general entropy production goes down.
- The micro-scale process that is a direct result of friction caused by the mechanical response of the surface engineered layer due to energy dissipation within the alternating nano-layers of the multilayered coatings [7] or within a layer of a High Entropy Alloy coating [8] or as an integrative mechanical response of the coating/carbide tool system [9]. Such processes develop gradually and also contribute to the reduction in entropy production.

In this review paper, the wear performance of a number of surface engineered tribo-systems is considered in relation to their adaptive response to external stimuli, based on several case studies.

**Figure 1:**
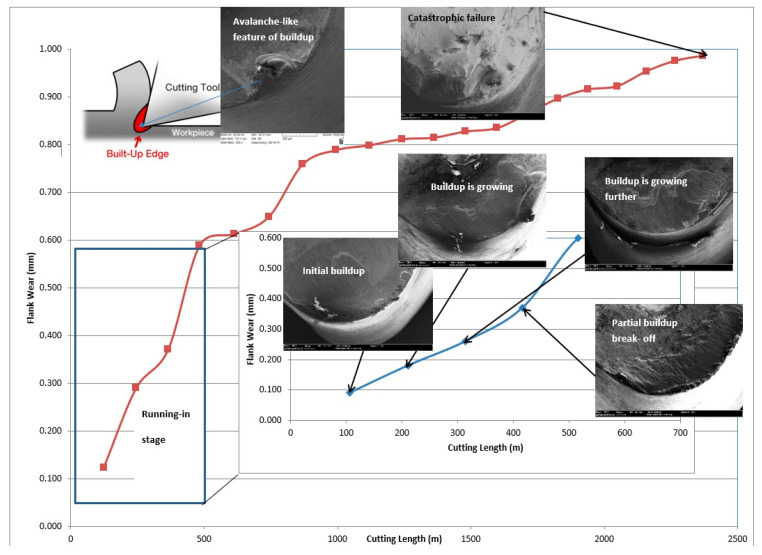
Schematic presentation of super duplex stainless steel (SDSS) machining with built-up edge formation using an uncoated cemented carbide cutting tool. Wear resistance data with progressive Scanning Electron Microscope (SEM) studies of built-up edge formation on the friction surface. Reproduced from [31].

This research chiefly focuses on extreme cutting conditions of hard to machine materials (such as stainless steels, Ti alloys, Compacted Graphitic Iron (CGI)) associated with strong built-up edge formation. Machining of hard-to-cut materials with intensive built-up edge formation is a very complex tribological phenomenon. Several simultaneous processes take place within the tribo-system during cutting under the outlined conditions [9]. The cutting tool is a critical part of the tribo-system that enables control over the overall frictional process [10]. For cemented carbide cutting tools which have the most widespread use with and without coatings, these processes are the following: (1) transfer of material from the counter body (chips and workpiece) to the surface of the tool; (2) intensive adhesion of the material to the surface that results in built-up edge formation (Figure 1); (3) interaction of adhered fragments of workpiece material with the surface of the tool as well as the (4) the environment and (5) complex tool/chip/workpiece interface phase transformations [11,12,13]. As was outlined above, the dynamically formed built-up layer consists of heavily deformed and refined particles of machining material, as well as various compounds generated during cutting. The built-up layer is similar in many ways to a composite material [10,11]. The “ceramic-like” built-up layer offers some protection to the tool surface. However, the stability of a built-up layer as a structure is very low, especially when the tool, composed of a cemented carbide is used under the outlined attrition wear conditions [12].

From a tribological viewpoint, machining of these materials proceeds under conditions of seizure at the tool/chip interface, which by itself, represents a catastrophic wear mode [10]. When certain hard-to-machine materials such as stainless steels are machined, the essential feature of chip formation is varying shear strength of the work material at the chip/tool interface: a phenomenon that is similar to stick–slip conditions [9]. The stick–slip phenomenon is related to self-organized critical processes [14,15,16,17,18] which occur during tribological processes in general [19,20] and metal cutting in particular [21].

Due to the cyclic growth, the built-up eventually begins to behave like an avalanche, leading to catastrophic failure of the entire tribo-system (Figure 1) [22,23,24,25]. Control over this process aims to reduce (a) the probability of occurrence and (b) the size of energy dissipation bursts, i.e., avalanches (built-ups) [23,24,25]. The dissipation of energy into a lower energy state in a self-organized critical system can be quite damaging when large avalanches cause a catastrophe of the entire system [23,24,25]. When the system’s energy is released through the triggering of smaller avalanches, the large avalanches are postponed or even potentially prevented [23,24,25,26,27]. For example, the generation of self-protective/lubricating tribo-films on the surface of specially designed adaptive PVD coatings could lead to the formation of small, unstable built-ups [28]. As they break away, energy in the system is dissipated through smaller-scale surface damaging processes. Such an outcome can be efficiently accomplished by the adaptive response of surface engineered tools. In this way, wear resistance of the system could be improved.

**Figure 2:**
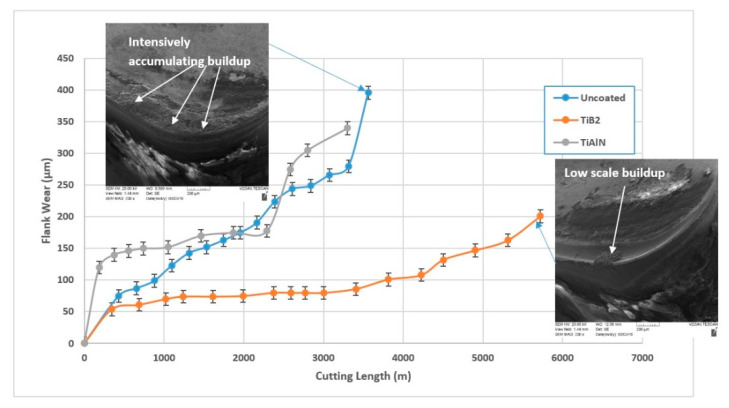
Tool life of TiB2 coated and uncoated tools after full-scale testing during rough turning of TiAl6V4 alloy. Reproduced from [28], with permission from Elsevier, 2017.

The discovery of self-organizing processes is one of the most significant recent achievements in fundamental science [29]. This concept is widely applied in the understanding of various processes in different fields of science and technology [30]). However, there exists a very limited amount of such studies related to contemporary thin-film coatings which function under strongly far from equilibrium tribological conditions with the extremely heavy loads and high-temperatures present in high-performance machining [10]. As such, the outlined case studies provide a perfect subject for the investigation of self-organizing processes. All the case studies presented below are based on the results of statistically validated cutting tests (see below Figures 1, 2, 4, 9, 10, 13 and 16) according to the general practice used in machining studies.

To understand this approach in greater detail, the wear performance of an uncoated cemented carbide cutting tool is considered first, during the machining of one of the most difficult to machine super duplex stainless steels (SDSS) (see Figure 1) [31]. Figure 1 shows that machining with a strong built-up edge is similar to avalanche-like behavior (see a schematic display with the built-up SEM image at the top left corner of Figure 1). Progressive SEM studies of the worn surface (Figure 1) show that the built-up generates, gradually accumulates and eventually breaks down during the running-in phase of wear. [31]. As cutting progresses, similar behavior occurs repeatedly. When a large sized built-up (avalanche) eventually breaks away, it can easily damage the edge of the tool and cause its failure (SEM image at the top of Figure 1).

Several coatings were developed with enhanced adaptive performance [26]. They are capable of presenting the adaptive response to external stimuli of the coated tool tribo-system at two major levels: the nanoscale and the micro-scale. A first group of coatings can protect and lubricate the surface during friction via the adaptive response at the nano-scale level, in particular through the formation of thermal barrier [27] or lubricating [28] tribo-films on the cutting tool surface. The other group of coatings can exhibit the adaptive response at the micro-scale level, in particular: (a) through the gradual flaking of the entire surface engineered layer [32] or (b) through the response of the whole coating/substrate tooling material [8].

The goal of this review paper is to demonstrate how control over the wear performance of coated cutting tools could be achieved through the adaptive response of various coating types to ensure superior wear performance of the surface engineered tribo-system under severe conditions prevalent during strong built-up edge formation.

## 2. Case Studies

### 2.1. Coatings That Produce Different Types of Tribo-Films during Cutting through the Adaptive Response of the Tribo-System at the Nano-Scale

#### 2.1.1. Coatings that Create a Layer of Lubricating Tribo-Films. Machining of a Ti Alloy with Strong Built-Up Edge (BUE) Formation

It is a significant challenge to develop a wear resistant PVD coating for the machining of Ti alloys. One of the main reasons for this is the intensive sticking of the titanium workpiece to the tool surface which quite often results in the detachment of the entire coatings layer [9]. Therefore, an uncoated tool often outperforms a coated one, such as the widely used AlTiN (Figure 2). An effective way to control built-up edge formation during the machining of Ti alloys is the application of self-lubricating adaptive coatings. Application of the boride family of coatings [33] presents a very promising avenue in this regard.

The detailed wear performance studies in this topic are performed for the self-lubricating TiB_2_ PVD coating [20]. Figure 2 presents the data concerning the wear resistance of the uncoated cutting tool and several coated tools during rough turning of a Ti alloy with intensive BUE formation [28].

**Figure 3:**
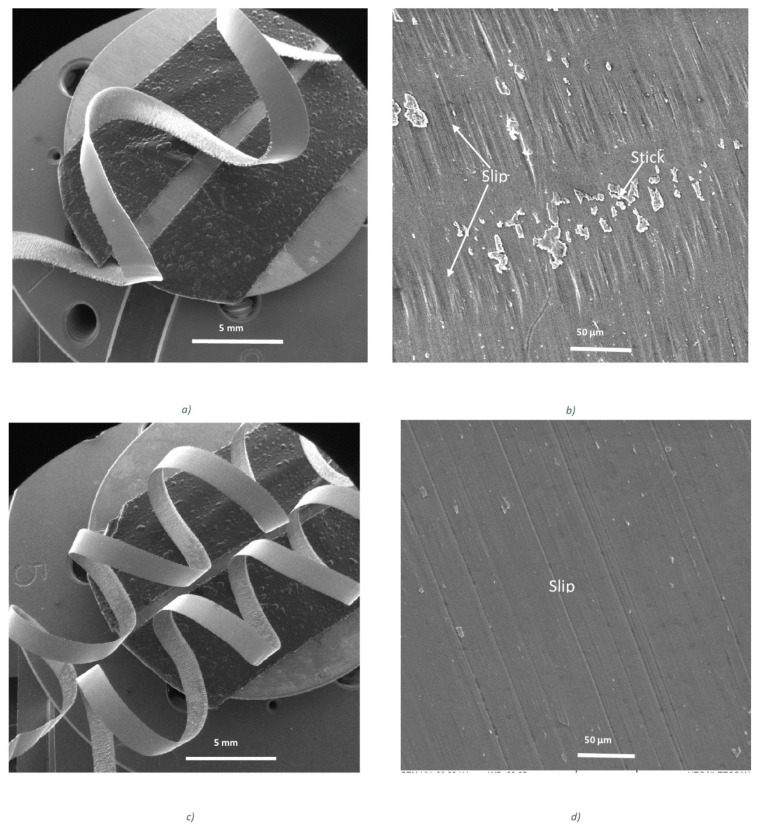
Type of chips and morphology of the chip undersurface for the (**a**,**b**) uncoated and (**c**,**d**) TiB2 coated cutting tools during rough turning of Ti6Al4V alloy. Reproduced from [28], with permission from Elsevier, 2017.

The results show that the wear performance of the uncoated tool is strongly surpassed by that of the TiB_2_ coating. (Figure 2). This is confirmed by SEM investigations of worn surfaces (Figure 2). SEM analysis indicates strong built-up formation in the uncoated tool and a very low-scale built-up in the TiB_2_ coated tool. Chip types and undersurface indicate different frictional phenomena occurring at the tool/chip interface. SEM images of the chip types and undersurfaces are presented in Figure 3.

They show that the typical stick–slip phenomenon [15,16] occurs on the undersurface of the chips formed by the uncoated tools (Figure 3b) [28]. This phenomenon is strongly diminished in the tool with a TiB_2_ self-lubricating coating. Different zones of stick and slip phenomena typical for the uncoated tool are not present there, and a very smooth chip undersurface forms (Figure 3d).

**Figure 4:**
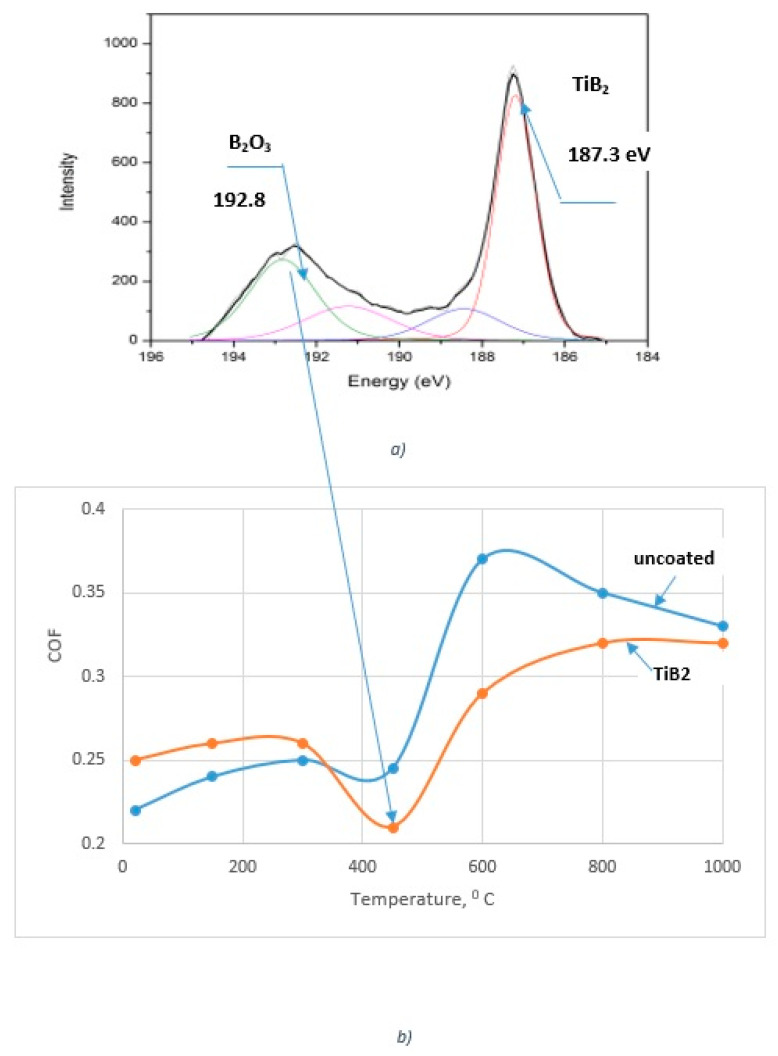
Worn surface of TiB2 coated inserts: (**a**) high resolution B1s X-ray Photoelectron spectrum related to (**b**) COF data vs. temperature. Reproduced from [28,36], with permission from Elsevier, 2017 and 2020.

XPS analysis of the worn surface indicates the formation of lubricating B_2_O_3_ tribo-films (Figure 4a). At temperatures of around 450–500 °C, this compound melts [34,35] and forms a liquid lubricant which greatly improves the frictional conditions. Coefficient of friction (COF) values drop at operating temperatures within 450–680 °C [36], Figure 4b. As a result, built-up size is greatly reduced (SEM images in Figure 2) and control over the built-up edge formations is thereby established.

#### 2.1.2. Coatings That Create a Layer of Thermal Barrier Tribo-Films

##### Machining of SDSS with Intensive Built-Up Edge Formation

**Figure 5:**
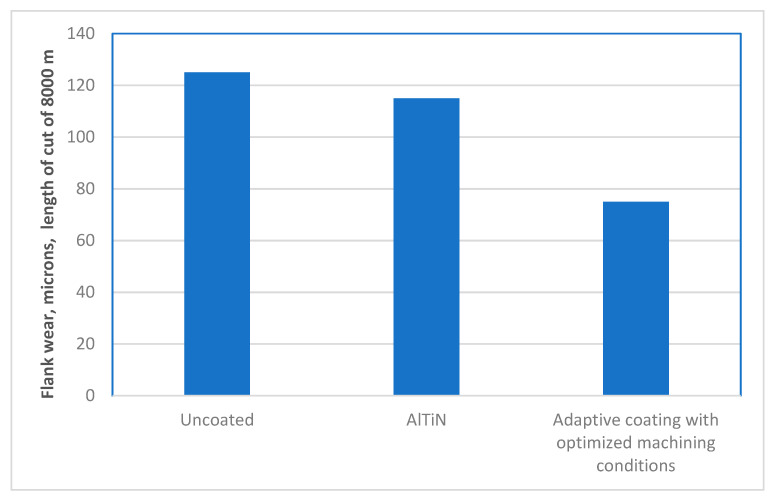
Tool life data of SDSS machining for the uncoated and various coated cutting tools [31].

**Figure 6:**
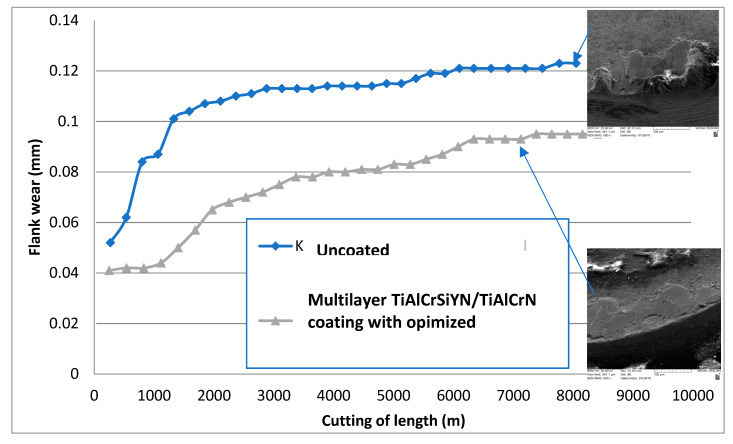
Wear resistance data (flank wear vs. length of cut) of SDSS machining for uncoated and PVD coated cutting tools with varying machining conditions. Reproduced from [31].

Wear performance of the coating that creates a thermal barrier nano-layer of tribo-films was investigated during the machining of super duplex stainless steel (SDSS). Figure 5 presents the results of wear resistance studies of the uncoated cutting and coated tools. The coatings tested were as follows: a benchmark AlTiN PVD coating widely used in industrial applications for stainless steel machining [31] and adaptive TiAlCrSiYN/TiAlCrN nano-multilayer coatings [31]. Adaptive coatings under optimized machining conditions have been shown to improve tool life up to 40% and up to 35% compared to the respective uncoated and AlTiN coated samples (see Figure 5). Figure 6 presents the flank wear vs. length of cut data for uncoated and coated cutting tools. The highest wear rate was observed on the uncoated insert. The SEM image of the cutting edge depicts the intensive formation of a built-up edge.

The application of an adaptive TiAlCrSiYN/TiAlCrN nano-multilayer coating under optimized machining conditions [37,38,39] significantly reduces the wear rate and leads to the formation of down-scaled built-ups that are smaller and thinner. (Figure 6).

**Figure 7:**
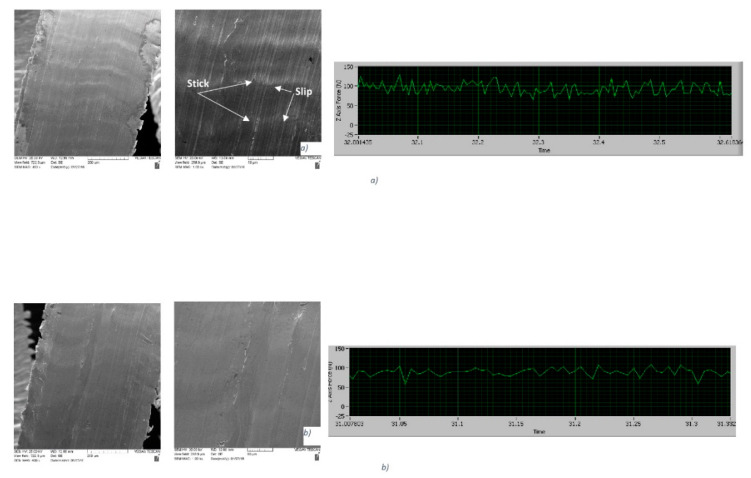
Chip undersurface at different magnifications (400× and 1000×) and cutting forces data: (**a**) uncoated tool; a (**b**) tool with a TiAl60CrSiYN/TiAlCrN multilayer coating, with optimized machining conditions. Reproduced from [31].

Chip undersurfaces are excellent fingerprints of the frictional surface phenomenon. SEM data of the chip undersurface were combined with frictional (cutting) force data at the very beginning of the process, as presented in Figure 7.

**Figure 8:**
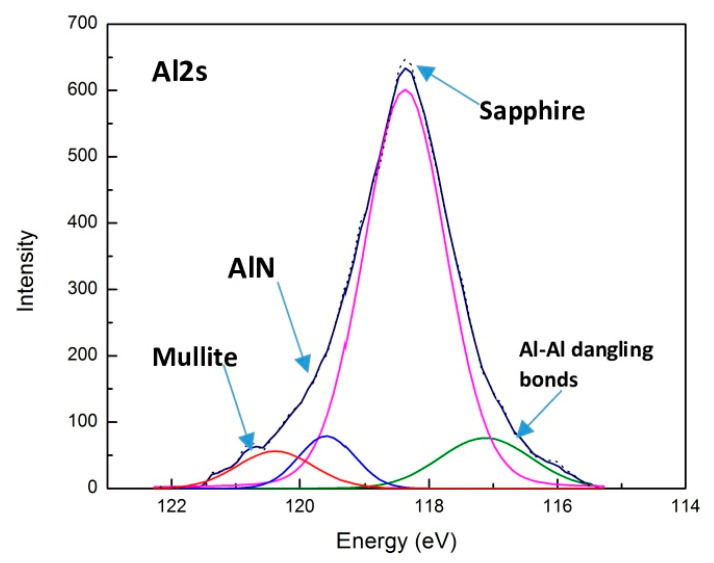
XPS data for tribo-films formed on the worn surface of multilayer TiAlCrSiYN/TiAlCrN thin film coating during running-in stage (after turning of 1000 m): Photoelectron Al2s spectra on the rake surface of the worn coated tool tested. Reproduced from [31].

SEM images show that the typical stick–slip phenomenon [21] occurs on the undersurface of the chips formed by uncoated tools (Figure 7a). Spikes of frictional forces are the most intensive in the uncoated cutting tool, which indicates that the stick–slip phenomenon (wave-like patterns on the chip undersurface indicated by arrows in Figure 7a), is directly related to the process of self-organized criticality [16,17]. This phenomenon is severely diminished in the coated tool—the wave-like patterns are practically eliminated on the chip undersurface (Figure 7b) and frictional force spikes are brief and seldom for the coated tools under optimized machining conditions (Figure 7b). This is a direct indication of a significant improvement in the frictional performance. Tribo-film formation on the friction surface that results from tribo-oxidation of the coating layer, plays a critical role in the adaptive response of the surface-engineered systems, especially under extreme frictional conditions [10]. Different types of tribo-films form on the surface of the cutting tools with TiAlCrSiYN/TiAlCrN multilayer coatings, such as sapphire and mullite thermal barrier ceramic films (Figure 8) [26,27,31].

A major feature of the TiAlCrSiYN/TiAlCrN adaptive coatings is their ability to form a nanoscale tribo-ceramic layer with an extremely high protective capacity, which radically alters the thermal properties of the friction surface [4,31]. This case study mainly illustrates the effects of protective, thermal barrier tribo-ceramics, which control the performance of the surface engineered layer [31]. They play an unprecedented role when the system operates under the severe conditions associated with strong built-up edge formation during SDSS machining. Figure 8 shows the photoelectron line Al2s components corresponding to the different types of aluminum chemical bonds in simple and complex oxide tribo-films and nitride coatings. The phase composition of aluminum-based compounds on the worn surface is regulated by chemical shifts in the Al2s component. Al-Si-O bonds are formed in the composite oxide with a mullite structure. Al-O bonds are typical of the Al_2_O_3_ composite and Al-N bonds are the residual parts of the initial nitride coating that were not oxidized during the cutting process. A certain amount of Al-Al dangling bonds was observed, indicating the amorphous-like layer structure [26]. All of this directly affects the tool life and wear performance (Figure 6), demonstrating the efficiency of the outlined BUE control strategy.

##### High Speed Machining of Ti Alloys: Combination of Built-Up Edge Formation and Cratering

Wear phenomena during the machining of a TiAl6V4 alloy are highly complex [10,13,28]. Intense adhesive interaction at the workpiece–tool interface occur in combination with built-up edge formation [28]. The built-up predominantly forms at lower speeds and partially protects the surface of the tool [28]. On the other hand, the built-up is an unstable structure with a temporal avalanche-like performance that eventually leads to severe surface damage once the structure is destroyed (turned off) during cutting [18]. Moreover, during the machining of Ti, a dynamically formed thermal barrier TiC interlayer is formed at the built-up–carbide tool interface [28]. However, the predominant wear mechanism of the cutting tool is significantly altered as the cutting speed increases [40]. The intensity of built-up edge formation is lower at higher speeds (built-up is intensively worn out) [40]. Therefore, the thermal barrier TiC layer is no longer capable of effectively performing its protective role at the tool–chip interface. This is combined with the rapid (thermally enhanced) diffusion of the cutting tool material into chips [40]. For this reason, the cratering wear mechanism determines the overall tool life under the outlined conditions.

In general, the CrN coating is a good candidate for non-ferrous alloy machining because it has a very low affinity with workpieces [41]. CrN is also known to have high chemical stability, resulting in good corrosion and oxidation resistance [42], tribological properties (low coefficient of friction), and toughness [43,44,45,46]. These properties are highly useful for the machining of sticky titanium alloys.

**Figure 9:**
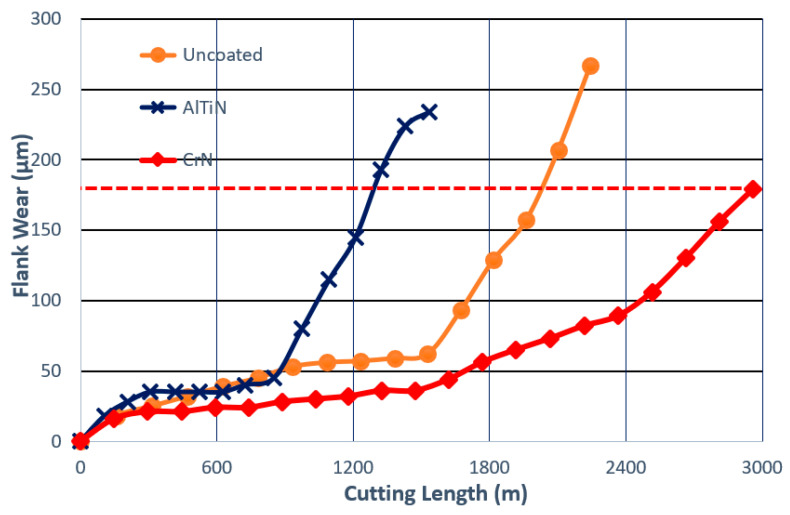
Flank wear vs. length of cut data under wet conditions at 150 m/min for coated and uncoated tools during finish turning of TiAl_6_V_4_ alloy. Reproduced from [36], with permission from Elsevier, 2020.

Tool life and wear performance studies were conducted at a cutting speed of 150 m/min where built-up formation is diminished, and tool life is short due to intensive crater wear [40]. Figure 9 presents flank wear vs. length of cut data for the wet turning of both coated and uncoated tools at a cutting speed of 150 m/min. The CrN coated tool outperforms the AlTiN coated tool by ~129% and the uncoated tool by ~45%.

Two wear mechanisms are prevalent for the outlined conditions: (1) crater wear combined with (2) intensive adhesive interaction at the tool–chip interface resulting in built-up edge formation. At elevated cutting speeds, crater wear predominates on the rake face due to the generation of high cutting temperatures [47]. Crater wear propagates overtime of the cutting, and rapid tool failure occurs when crater wear expands to the flank edge of the cutting tool.

**Figure 10:**
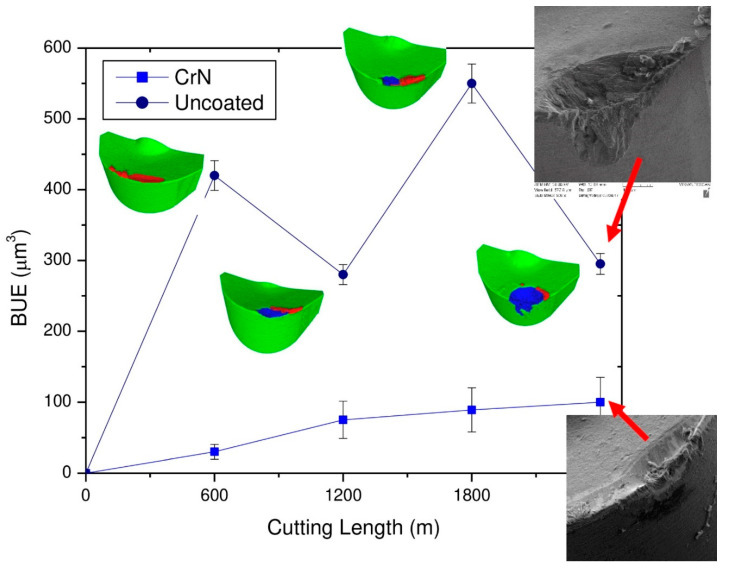
3D volume numerical data showing built-up for coated and uncoated tools considering the volume of peaks above the reference surface of the original tool during finish turning of TiAl_6_V_4_ alloy. Red areas are built-ups, and blue are cratering areas. Reproduced from [36], with permission from Elsevier, 2020.

Figure 10 shows that machining with built-up edge formation features similarities to the behavior of an avalanche. 3D progressive studies of wear show that the built-up periodically forms, accumulates and breaks down [31]. This behavior occurs in combination with intensive cratering during the ongoing cutting process.

**Figure 11:**
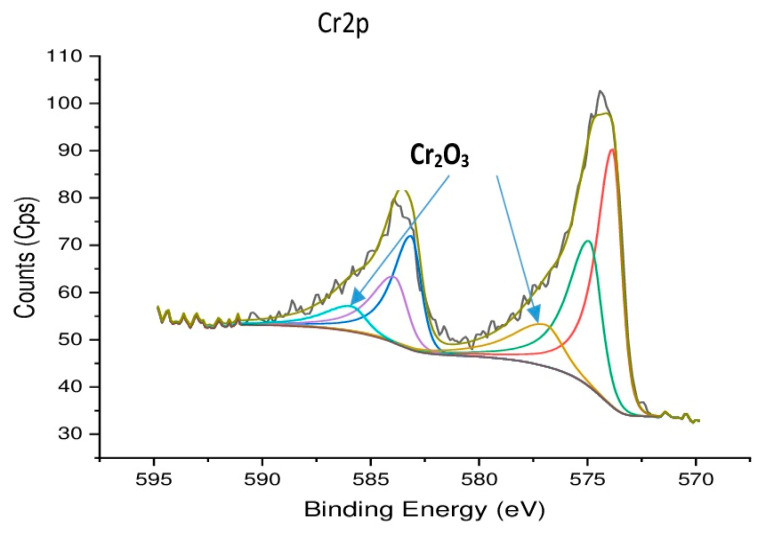
High-resolution XPS data of worn rake surface of CrN coated insert, Cr2p spectrum. Reproduced from [36], with permission from Elsevier, 2020.

The tribological performance of the tool/chip tribo-system is strongly related to the adaptive response of the coating layer. This is due to the formation of tribo-films on the friction surface [13]. During high-speed cutting, a strong temperature gradient is created with the highest temperature being present on the rake surface [31] of the tool where crater wear is most intensely propagating. Investigation of tribo-film formation on the worn surface of the tool through XPS analysis helps explain the superior performance of the CrN coating (Figure 11).

The obtained data show that the CrN coating layer tribo-oxidizes, forming a Cr_2_O_3_ tribo-ceramic layer on the rake surface of the tool. These films have a corundum structure [48] and provide thermal barrier properties at high temperatures of cutting [49]. The temperature at the tool–chip interface under the outlined conditions is around 900–950 °C [40], at which these tribo-films perform fairly effectively. This can indirectly, by function, replace the thermal barrier TiC interlayer that had formed at lower speeds [50] and helps to reduce crater wear intensity, thereby improving tool life. Due to the enhanced ability of the CrN coating to form thermal barrier Cr_2_O_3_ tribo-films under operation, the intensity of crater wear is strongly diminished and tool life is increased (Figure 9).

**Figure 12:**
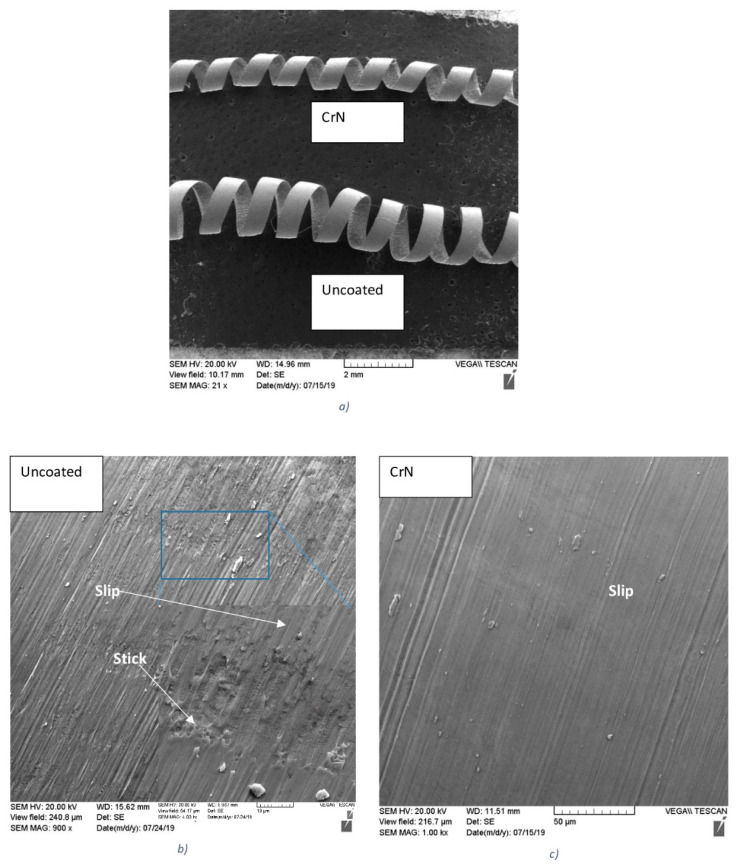
Type of chips (**a**) and morphology of the chip undersurface for the—(**b**) uncoated and (**c**) CrN coated cutting tools during machining of Ti_6_Al_4_V alloy.

This also substantially improves the tribological performance, resulting in the formation of more curled chips in the tool with a CrN coating (Figure 12a). The chips on the uncoated tool clearly indicated a stick-slip phenomenon on their undersurface (Figure 12b). In chips formed by CrN coated tools, the undersurface morphology was smoother and mostly slipping was observed.

As a result, built-up size is significantly reduced (SEM images in Figure 10), and the wear performance is brought under control.

### 2.2. Coatings That Create an Adaptive Response at the Micro-Scale through Partial Flaking of the Surface Engineered Layer

**Figure 13:**
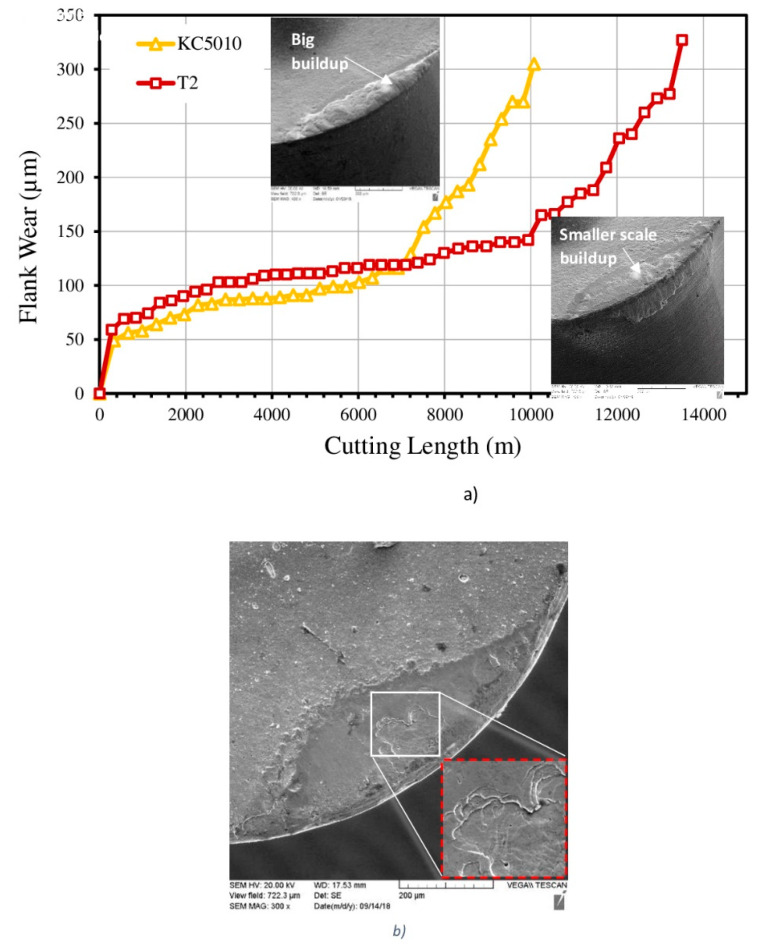
Flank wear versus cutting length for different PVD coatings during machining of compacted graphitic iron (CGI): (**a**) flank wear data combined with SEM images of built-up; (**b**) SEM data after builtup removal (by chemical etching). Reproduced from [32], with permission from Elsevier, 2019.

The tool life during machining of a compacted graphite iron (CGI) tool was analyzed under the extreme conditions of dry (coolant-free) turning. Figure 13 shows the tool life figures of the benchmark Al_60_Ti_40_N (KC 5010) arc PVD coating and the novel, low stress multilayer superfine cathode (SFC) PVD coating (T2) with the same composition [51]. The new coating achieved a ~35% tool life improvement over the benchmark coating, due to the the significantly lower intensity of built-up edge formation (see SEM images in Figure 13a). This can be attributed to the top surface of the coating undergoing gradual flaking [51] instead of the entire coating layer being fractured in depth (Figure 13b).

**Figure 14:**
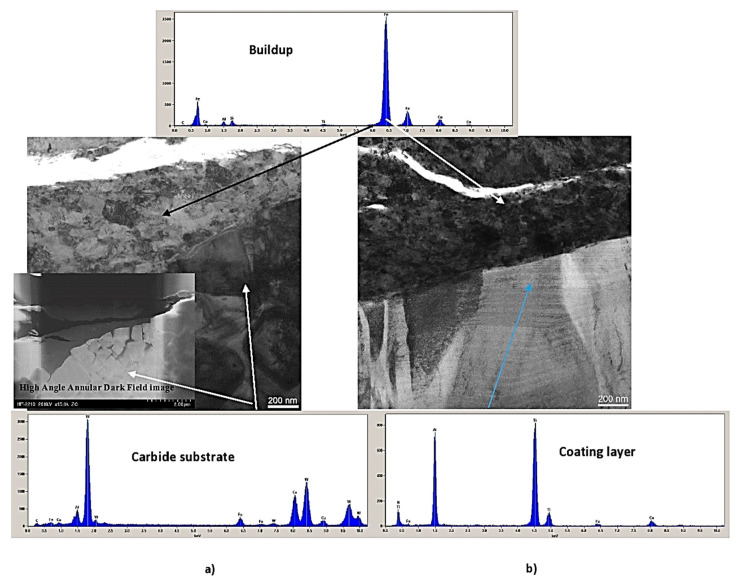
Cross-sectional TEM images of the rake face inserts: (**a**) AlTiN-benchmark coating with, High Angle Annular Dark Field (HAADF) magnified TEM image of (**b**) SFC-AlTiN 10um; length of cut of 400 m. Reproduced from [32], with permission from Elsevier, 2019.

Cross-sectional TEM images of the worn area (Figure 14) show that the built-up had formed on the rake face of the turning coated inserts after a length of cut of 400 m.

As a result of this interaction, the entire layer of the AlTiN (benchmark) coating had worn out (destroyed) and the built-up formed directly on the WC-Co substrate (Figure 14a). However, in the case of the SFC–AlTiN multilayer, the coating layer is still clearly present (Figure 14b). A High Angle Annular Dark Field (HAADF) image of the same area (Figure 14a) shows that the built-up had directly formed on the WC-Co substrate, where heavily deformed large WC grains are visible in the benchmark coating. There are no visible WC grains in the SFC–AlTiN multilayer coating and the built-up has formed directly on the coating layer. (Figure 14b). The magnified TEM image (Figure 14b) confirms that the built-up/coating interface is sharp and no interlayer has formed on it. Most importantly, no deformation (bending) of the AlTiN coating columnar structure can be observed, which is typical for a coating that cannot withstand high frictional forces during cutting.

Figure 14 shows that due to the adaptive response of the surface engineered layer (through partial flaking, Figure 13) the size of the energy dissipation burst of the self-organized critical systems (built-ups) becomes significantly lower. In this way, large avalanches (Figure 14a) are prevented, and surface damage is substantially reduced (Figure 14b).

**Figure 15:**
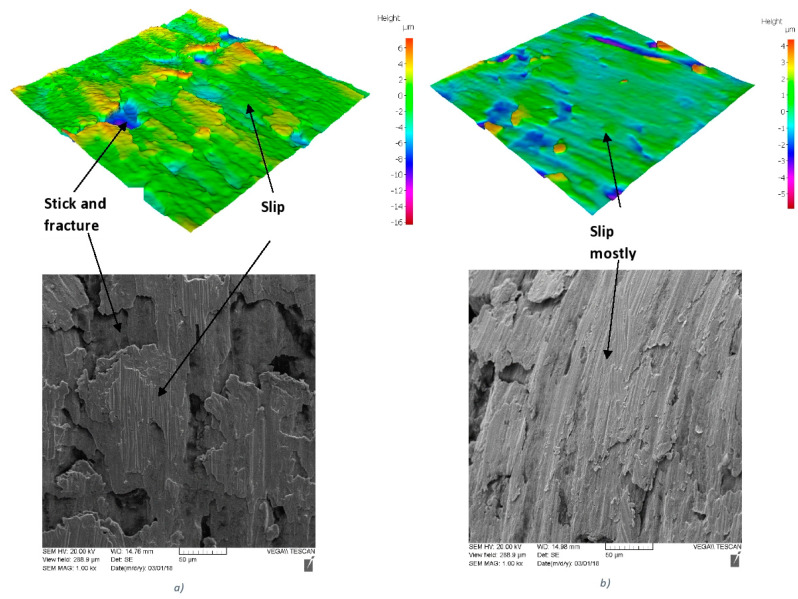
SEM images of chip undersurface combined surface texture obtained by Infinite Focus G5 focus variation microscope (Alicona) with for (**a**) regular PVD arc (benchmark) coating and (**b**) low-stress TiAlN multilayer coating.

An analysis of the chip undersurface was made for the benchmark and the novel low-stress coated cutting tools (Figure 15). Intensive stick–slip phenomena combined with deep surface damage is typical for the benchmark coating (Figure 15a). In the new low-stress coating, the intensity of this catastrophic phenomenon is greatly reduced—mostly slip areas are visible (Figure 15b).

### 2.3. Surface Engineered Layer That Demonstrates the Adaptive Response of the Entire Coated Carbide Tool System

The tool/chip/workpiece is a tribo-system (or structure) whose constituent parts are interconnected or functionally integrated. The term “structure” is used here in the generic sense [18]. A failure of one part may cause the entire structure to fall apart as well. An integrative approach is critically needed to control such complex engineering systems (structures) [18].

**Figure 16:**
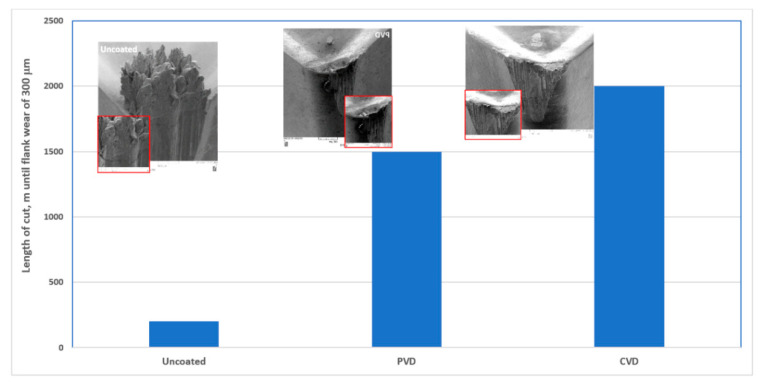
Wear performance data on SS 304 machining with built-up edge formation on the friction surface of coated carbide tools: flank wear vs. length of cut, m with Scanning Electron Microscope (SEM) studies of the worn surface at the end of tool life. Reproduced from [9], with permission from Elsevier, 2020.

Results of flank wear and SEM studies of built-up formation in the two combinations of coated carbide tools in comparison with the uncoated tool, are presented in Figure 16.

The results presented show the growth and further detachment of the built-ups which indicate self-organized critical performance that is similar to avalanches [18]. As can be seen in Figure 16, this eventually leads to the catastrophic failure of the entire tribo-system (SEM images, Figure 16).

It was demonstrated that superior wear performance and longer tool life can be attributed to a surface engineered carbide material with an optimal combination of the coating/substrate properties as a whole [8]. Such a structure has the potential to present adaptive response on two scales: nano- and micro. Under severe frictional conditions associated with self-organized critical performance (i.e., strong built-up edge formation), the mechanical properties which determine the overall resistance of the coated carbide tool to severe external impact have a direct effect on the length of tool life (Table 1) [8].

The fracture toughness [52,53] of two different coating systems is shown in Table 1. The toughness of the PVD coating on the fine-grained carbide substrate is 1.35 N/μm, whereas the toughness of the CVD coating on the medium-grain carbide substrate is greater (2 N/μm). In the PVD coated carbide tool, the substrate hardness is 22.6 GPa and the coating hardness is 30 GPa. In the bi-layer CVD coating; however, the hardness of the Al-oxide outer layer is 33 GPa and the hardness of the TiCN sublayer is 30 GPa. The hardness of the CVD coated substrate is 16.8 GPa. As shown in Table 1, the lower Co content of the PVD coated carbide alloy contributes to the higher hardness. Conversely, a higher Co content leads to lower carbide material hardness, which could in turn improve the toughness. This should be considered in combination with plasticity index (PI) values (Table 1) [54]. Higher values of PI are associated with higher energy dissipation under loading [54]. The more energy becomes dissipated, the less of it is spent on deformation and damage of the carbide. The amount of energy is insufficient to begin the deformation of the carbide material. Both of these parameters could be used in a complementary fashion to determine the wear behavior of heavily loaded tribo-systems under conditions of unstable attrition wear which result in strong built-up edge formation. Therefore, a CVD coated carbide material with lower hardness and higher PI and toughness, exhibits superior wear performance as well as longer tool life.

**Figure 17:**
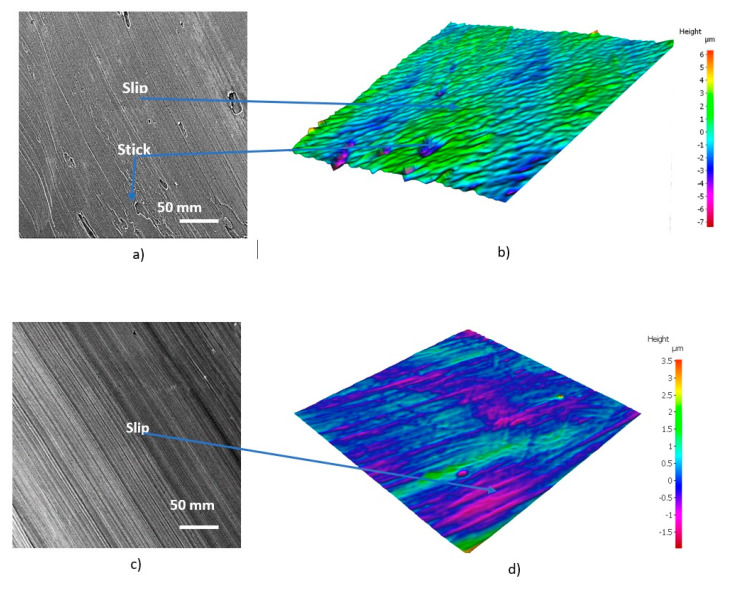

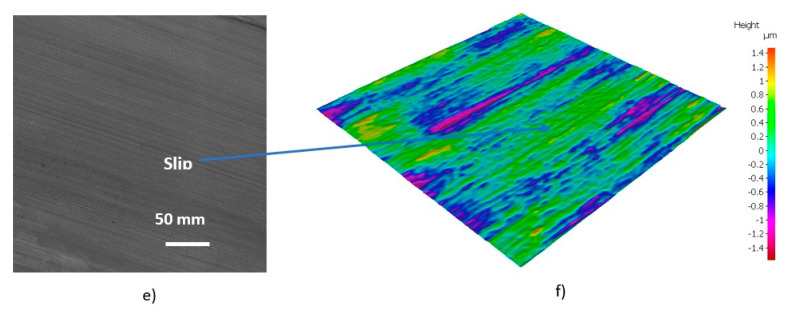
SEM images of chip undersurface combined surface texture obtained by Infinite Focus G5 focus variation microscope (Alicona) with for (**a**,**b**) uncoated too; (**c**,**d**) PVD coated tool; (**e**,**f**) CVD coated tool. Reproduced from [9], with permission from Elsevier, 2020.

Stick–slip wear patterns observed on the chip undersurface were produced by the uncoated tool (Figure 17a,b). However, no sticking is present on the chip undersurface generated by the PVD coated tool, but the slip is severe (Figure 17c,d). In the CVD coated tool, only a mild slip pattern can be observed (Figure 17e,f). It can be concluded that tribological conditions are strongly improved at the tool/chip interface and the self-organized critical (SOC) process is brought under control in the CVD coated carbide material with the optimal combination of micro-mechanical properties. This is a direct indication of integrative system behavior.

## 3. Future Research Directions for Achieving Better Control over Wear Performance during Machining with BUE Formation: Multi-Functional Coatings

Up to now, the majority of the PVD coatings used in cutting tool applications belong to the category of nitride-based coatings which contain a limited number of alloying elements (usually 2–3). However, a promising new group of coatings has been recently introduced, which are multifunctional coatings [55]. These coatings are able to address various wear mechanisms, thereby further improving wear resistance [33]. Two major categories of multifunctional coatings show strong potential in achieving control over severe BUE formation during machining. The first is the boride family of coatings, which possess high hardness and chemical stability of the transition metal borides [33,56,57,58,59,60,61,62]. During cutting they also exhibit self-lubricating/protective properties [28]. The second type is high entropy alloy coatings [63], whose combination of protective/lubrication characteristics with beneficial micro-mechanical properties is responsible for their superior wear performance during machining with BUE formation.

### 3.1. Multifunctional Boride Coatings

At the moment, the most widely used boride coating for cutting tool applications is TiB_2_. As was mentioned previously, this coating is highly efficient in the controlling of BUE formation during Ti alloy machining [28]. However, a wide variety of transition metal-based boride coatings has recently appeared on the market [33,56]. In addition to the beneficial micro-mechanical properties [58,59,60,61,62] the major advantage of these coatings is their strong potential to deliver multi-functional performance due to the formation of various tribo-films under operation. This provides extra opportunities to control BUE formation in a several ways, such as the improvement of thermal barrier and lubricating properties of the coated tools.

### 3.2. High Entropy Alloyed Coatings (HEAC)

In contrast to typical PVD coatings, which mostly contain no more than three alloying elements responsible for their basic properties, the high entropy alloy coatings contain five and more alloying elements [63]. In contrast to the TiAlCrSiYN/TiAlCrN multilayer coating presented in [31], which is designed on the principle of finding the of a coating’s emergent characteristics, HEAC are developed using a different basis, when five or more alloying elements are present in equal or relatively large proportions [63]. The term “high-entropy alloys” was introduced because the entropy increase in mixing is substantially higher when there is a greater number of elements in the composition and when their proportions are more or less equal [63]. Such coatings exhibit improved frictional and micro-mechanical properties, such as the combination of high hardness and improved toughness as well as high thermal and oxidation stability [64,65,66,67,68,69,70,71,72,73,74,75,76]. Therefore, they have the potential, on one hand, to form several beneficial tribo-films (protective and lubricating) on the friction surface and, on the other hand, provide energy dissipation within the body of the coating layer, with the goal of reducing the surface damage caused by the detachment of built-up. The amount of published works concerning the wear performance of HEAC during cutting is limited [77,78] and these publications lack in-depth studies of their wear performance. As such, this presents a promising subject for future detailed research in the outlined direction.

## 4. Conclusions

Machining of various hard to machine materials usually occurs alongside intense built-up edge (BUE) formation. Metal cutting with BUE formation is associated with the stick–slip phenomenon at the tool/chip interface and constitutes a self-organized critical (SOC) process. Since the built-up edge is a semi-protective, very low-stability structure, it generally acts like an avalanche that eventually results in the catastrophic failure of the entire cutting tool/workpiece tribo-system. To address this issue, it is of primary importance to bring the self-organized critical processes under control. The chief goal of this control is to reduce (a) the probability of occurrence and (b) size of avalanches (built-ups). In this way, the large avalanches are postponed and even possibly prevented. One of the most efficient ways to implement such a strategy is through the adaptive response of the surface-engineered cutting tools. Several PVD and CVD coatings can be used to accomplish this task. These different coatings are designed to control built-up formation at different scales in a number of ways that can lead to improved wear performance. Such coatings can be classified into two categories: those that enhance the adaptive response at the nanoscale by providing self-lubricating and thermal barrier capacities, and those that produce an adaptive response at the micro-scale via the partial flaking of the coating layer. An integrative approach to coating/substrate design was also considered in this study. Other groups of coatings also demonstrate a strong potential to effectively control BUE formation. Multifunctional boride coatings can reduce the intensity of built-up edge formation via the generation of various tribo-films with protective and lubricating abilities. High entropy alloy coatings can also produce an adaptive response at two scales: nanoscale via formation of several beneficial tribo-films and simultaneously provide energy dissipation at the micro-scale within the body of the coating layer. Both of these processes act together to reduce surface damage once the built-up becomes detached from the surface of the tool.

## Figures and Tables

**Table 1:** Composition, structural characteristics and micro-mechanical characteristics for the coatings/carbide substrates [9]. (Reproduced from [9], with permission from publisher Elsevier, 2019.).

Coated Carbide Tool Material	Chemical Composition, Wt.%	Structural Characteristics	Hardness, GPa	Elastic Modulus, GPa	H/E	Plasticity Index (PI)	Fracture Toughness, N/μm (Load 100 N)
Physical vapor deposition (PVD) coated							
Carbide substrate material	WC-92.5 Co-7.5	Fine-grained cemented carbide	22.6	550	0.0414	0.635	
Coating layer	Al_60_Ti_40_N	Nano-crystalline	30	360	0.083		1.35
Chemical vapor deposition (CVD) coated							
Carbide substrate material	WC-87.3 Co-10 TiC-2.7	Medium-grained cemented carbide	16.8	538.7	0.0325	0.692	
Bi-layer coating:	Al_2_O_3_ top layer TiCN sublayer		33 30	390 420	0.085 0.071		2.0

## References

[1] Dimova E.G., Bryant P.E., Chankova S.G. (2008). Adaptive response: Some underlying mechanisms and open questions. Genet. Mol. Biol..

[2] Landini P., Volkert M.R. (2000). Regulatory Responses of the Adaptive Response to Alkylation Damage: A Simple Regulon with Complex Regulatory Features. J. Bacteriol..

[3] Wang I., Bisoyi H.K., Zheng Z., Gutierrez-Cuevas K.G. (2017). Stimuli-directed self-organized chiral superstructures for adaptive windows enabled by mesogen-functionalized grapheme. Mater. Today.

[4] Fox-Rabinovich G.S., Gershman I.S., Veldhuis S. (2020). Thin-Film PVD Coating Metamaterials Exhibiting Similarities to Natural Processes under Extreme Tribological Conditions. Nanomaterials.

[5] Gershman I.S., Gershman E.I., Mironov A.E., German S., Fox-Rabinovich G.S., Veldhuis S.C. (2016). Application of the Self-Organization Phenomenon in the Development of Wear Resistant Materials—A Review. Entropy.

[6] Fox-Rabinovich G., Totten G.E. (2006). Self-Organization during Friction: Advanced Surface-Engineered Materials and Systems Design.

[7] Yau B.-S., Huang J.-L., Lu H.-H., Sajgalik P. (2005). Investigation of nanocrystal-(Ti_1−*x*_Al*_x_*)N*_y_*/amorphous-Si_3_N_4_ nanolaminate films. Surf. Coat. Technol..

[8] Wang Z., Wang C., Zhao Y.L., Hsu Y.C., Li C.L. (2020). High hardness and fatigue resistance of CoCrFeMnNi high entropy alloy films with ultrahigh-density nanotwins. Int. J. Plast..

[9] He Q., Paiva J.M., Kohlscheen J., Beake B.D., Veldhuis S.C. (2020). An integrative approach to coating/carbide substrate design of CVD and PVD coated cutting tools during the machining of austenitic stainless steel. Ceram. Int..

[10] Trent E.M., Wright P.K. (2000). Metal Cutting.

[11] Kabaldin Y.G., Kojevnikov N.V., Kravchuk K.V. (1990). HSS cutting tool wear resistance study. J. Frict. Wear.

[12] Ahmed Y.S., Paiva J.M., Bose B., Veldhuis S. (2019). New observations on built-up edge structures for improving machining performance during the cutting of superduplex stainless steel. Tribol. Int..

[13] Fox-Rabinovich G.S., Kovalev A.I., Fox-Rabinovich G.S., Totten G. (2006). Self-Organization and Structural Adaptation during Cutting and Stamping Operations. Self-Organization during Friction: Advance Surface Engineered Materials and Systems Design.

[14] Feder H.J.S., Feder J. (1991). Self-Organized Criticality in a Stick-Slip Process. Phys. Rev. Lett..

[15] Zypman F., Ferrente J., Jansen M., Scanlon K., Abel P. (2003). Evidence of self-organized criticality in dry sliding friction. J. Phys. Condens. Matter.

[16] Bak P., Tang C., Wiesenfeld K. (1988). Self-organized criticality. Phys. Rev. A.

[17] Bak P., Tang C. (1989). Earthquakes as a self-organized critical phenomenon. J. Geophys. Res. B.

[18] Bak P. (1999). How Nature Works: The Science of Self-Organized Criticality.

[19] Nosonovsky M. (2010). Self-organization at the frictional interface for green tribology. Philos. Trans. R. Soc. A.

[20] Nosonovsky M., Bhushan B. (2009). Thermodynamics of surface degradation, self-organization and self-healing for biomimetic surfaces. Philos. Trans. R. Soc. A.

[21] Wang Q., Lu C., Ye G.G., Dai L.H. (2015). Modeling the tunned criticality in stick-slip friction during metal cutting. Model. Simul. Mater. Sci. Eng..

[22] Hoffmann H., Payton D.W. (2018). Optimization by Self-organized criticality. Sci. Rep..

[23] Cajueiro D.O., Andrade R.F.S. (2010). Controlling self-organized criticality in sandpile models. Phys. Rev. E.

[24] Cajueiro D.O., Andrade R.F.S. (2010). Controlling self-organized criticality in complex networks. Eur. Phys. J. B.

[25] Cajueiro D.O., Andrade R.F.S. (2010). Dynamical programming approach for controlling the directed Abelian Dhar-Ramaswamy model. Phys. Rev. E.

[26] Fox-Rabinovich G.S., Yamamoto K., Beake B.D., Gershman I.S., Kovalev A.I., Aguirre M.H., Veldhuis S.C., Dosbaeva G., Endrino J.L. (2012). Hierarchical adaptive nano-structured PVD coatings for extreme tribological applications: The quest for non-equilibrium states and emergent behavior. Sci. Technol. Adv. Mater..

[27] Beake B.D., Fox-Rabinovich G.S., Losset Y., Yamamoto K., Agguire M.H., Veldhuis S.C., Endrino J.L., Kovalev A.I. (2012). Why can TiAlCrSiYN-based adaptive coatings deliver exceptional performance under extreme frictional conditions?. Faraday Discuss..

[28] Chowdhury M.S.I., Chowdhury S., Yamamoto K., Beake B.D., Bose B., Elfizy A., Covelli D., Dosbaeva G., Aramesh M., Fox-Rabinovich G.S. (2017). Wear behavior of coated carbide tools during machining of Ti6Al4V aerospace alloy associated with strong built-up edge formation. Surf. Coat. Technol..

[29] Prigogine I. (1978). Time, Structure, and Fluctuations. Science.

[30] Oddershede L., Dimon P., Bohr J. (1993). Self-organized criticality in fragmenting. Phys. Rev. Lett..

[31] Fox-Rabinovich G., Paiva J.M., Gershman I., Aramesh M., Covelli D., Yamamoto K., Dosbaeva G., Veldhuis S. (2016). Control of Self-Organized Criticality through Adaptive Behavior of Nano-Structured Thin Film Coatings. Entropy.

[32] Abdoos M., Yamamoto K., Bose B., Fox-Rabinovich G., Veldhuis S. (2019). Effect of coating thickness on the tool wear performance of low-stress TiAlN PVD coating during turning of compacted graphite iron (CGI). Wear.

[33] Mitterer C. (1997). Borides in Thin Film Technology. J. Solid State Chem..

[34] Aouadi S.M. (2014). Lubricious oxide coatings for extreme temperature applications: A review. Surf. Coat. Technol..

[35] Mackenzie J.D., Claussen W.F. (1961). Crystallization and Phase Relations of Boron Trioxide at High Pressures. J. Am. Ceram. Soc..

[36] Chowdhury M.S.I., Bose B., Yamamoto K., Shuster L.S., Paiva J., Fox-Rabinovich G.S., Veldhuis S.C. (2020). Wear performance investigation of PVD coated and uncoated carbide tools during high-speed machining of TiAl6V4 aerospace alloy. Wear.

[37] Fox-Rabinovich G.S., Beake B.D., Yamamoto K., Aguirre M.H., Veldhuis S.C., Dosbaeva G., Elfizy A., Biksa A., Shuster L.S. (2010). Structure, properties and wear performance of nano-multilayered TiAlCrSiYN/TiAlCrN coatings during machining of Ni-based aerospace superalloys. Surf. Coat. Technol..

[38] Fox-Rabinovich G., Kovalev A., Veldhuis S., Yamamoto K., Endrino J.L., Gershman I.S., Rashkovskiy A., Aguirre M.H., Wainstein D.L. (2015). Spatio-Temporal Behavior of Atomic-Scale Tribo-Ceramic Films in Adaptive Surface Engineered Nano-Materials.

[39] Yuan J., Yamamoto K., Covelli D., Tauhiduzzaman M., Arif T., Gershman I., Veldhuis S., Fox-Rabinovich G. (2016). Tribo-films control in adaptive TiAlCrSiYN/TiAlCrN multilayer PVD coating by accelerating the initial machining conditions. Surf. Coat. Technol..

[40] Paiva J.M., Shalaby M.A.M., Chowdhury M., Shuster L., Chertovskikh S., Covelli D., Junior E.L., Stolf P., Elfizy A., Bork C.A.S. (2017). Tribological and wear performance of carbide tools with TiB2 PVD coating under varying machining conditions of TiAl6V4 aerospace alloy. Coatings.

[41] Lupicka O., Warcholinski B. (2017). The adhesion of CrN thin films deposited on modified 42CrMo4 steel. Ann. Mater. Sci. Eng..

[42] Milosev I., Strehblow H.H., Navinsek B. (1997). Comparison of TiN, ZrN, and CrN coatings under oxidation. Thin Solid Films.

[43] Berg G., Friedrich C., Broszeit E., Berger C. (1996). Development of chromium nitride coatings substituting titanium nitride. Surf. Coat. Technol..

[44] Sato T., Tada O., Hoke K., Besshi T. (1994). A crossed-cylinders testing for evaluation of wear and tribological properties of coated tools. Wear.

[45] Navinsek B., Panjan P., Milosev I. (1999). PVD coatings as an environmentally clean alternative to electroplating and electroless processes. Surf. Coat. Technol..

[46] Bertrand G., Mahdjoub H., Meunier C. (2000). A study of the corrosion behaviour and protective quality of sputtered chromium nitride coatings. Surf. Coat. Technol..

[47] Hatt O., Crawforth P., Jackson M. (2017). On the mechanism of tool crater wear during titanium alloy machining. Wear.

[48] Kramer A., Bignardi L., Lacovig P., Lizzit S., Batzill M. (2018). Comparison of surface structures of corundum Cr2O3 (0 0 0 1) and V2O3 (0 0 0 1) ultrathin films by x-ray photoelectron diffraction. J. Phys. Condens. Matter.

[49] Wang D.Y., Lin J.H., Ho W.Y. (1998). Study on chromium oxide synthesized by unbalanced magnetron sputtering. Thin Solid Films.

[50] Taylor R.E. (1961). Thermal conductivity of titanium carbide at high temperatures. J. Am. Ceram. Soc..

[51] Holmberg K., Laukkanen A., Ronkainen H., Wallin K., Varjus S. (2003). A model for stresses, crack generation and fracture toughness calculation in scratched TiN-coated steel surfaces. Wear.

[52] Sebastiani M., Johanns K.E., Herbert E.G. (2015). Measurement of fracture toughness by nanoindentation methods: Recent advances and future challenges. Curr. Opin. Solid State Mater. Sci..

[53] Ast J., Ghidelli M., Durst K., Göken M., Sebastiani M., Korsunsky A.M. (2019). A review of experimental approaches to fracture toughness evaluation at the micro-scale. Mater. Des..

[54] Fox-Rabinovich G.S., Veldhuis S.C., Skvortsov V.N., Shuster L.S.H., Dosbaeva G.K., Migranov M.S. (2004). Elastic and plastic work of indentation as a characteristic of wear behavior for cutting tools with nitride PVD coatings. Thin Solid Films.

[55] Kathrein M., Michotte C., Penoy M., Polcik P., Mitterer C. (2005). Multifunctional multi-component PVD coatings for cutting tools. Surf. Coat. Technol..

[56] Hale T., Lueth R. (1981). Boride Coated Cemented Carbide. U.S. Patent US4268582A.

[57] Dearnley P., Schellewald M., Dahm K. (2005). Characterisation and wear response of metal-boride coated WC–Co. Wear.

[58] Choi H.S., Park B., Lee J.J. (2007). CrB_2_ coatings deposited by inductively coupled plasma assisted DC magnetron sputtering. Surf. Coat. Technol..

[59] Bedse R.D., Sonber J.K., Sairam K., Murthy T.S.R., Hubli R.C. (2015). Processing and Characterization of CrB2-Based Novel Composites. High Temp. Mater. Proc..

[60] Audronis M., Kelly P.J., Arnell R.D., Valiulis A.V. (2006). Pulsed magnetron sputtering of chromium boride films from loose powder targets. Surf. Coat. Technol..

[61] Audronis M., Kelly P.J., Arnell R.D., Leyland A., Matthews A. (2005). Deposition of ulticomponent chromium boride-based coatings by pulsed magnetron sputtering of powder targets. Surf. Coat. Technol..

[62] Kiryukhantsev-Korneev P.V., Horwat D., Pierson J.F., Levashov E.A. (2014). Comparative Analysis of Cr–B Coatings Deposited by Magnetron Sputtering in DC and HIPIMS Modes. Tech. Phys. Lett..

[63] Gao M.C., Yeh J., Liaw P., Zhang Y. (2016). High-Entropy Alloys. Fundamentals and Applications.

[64] Hsieh M.-H., Tsai M.-H., Shen W.-J., Yeh J.-W. (2013). Structure and properties of two Al–Cr–Nb–Si–Ti high-entropy nitride coatings. Surf. Coat. Technol..

[65] Mayrhofer P.H., Kirnbauer A., Ertelthaler P.H., Koller C.M. (2018). High-entropy ceramic thin films: A case study on transition metal diborides. Scr. Mater..

[66] Pogrebnjak A.D., Bagdasaryan A.A., Yakushchenko I.V., Beresnev V.M. (2014). The structure and properties of high-entropy alloys and nitride coatings based on them. Russ. Chem. Rev..

[67] Lai C.H., Cheng K.H., Lin S.J., Yeh J.W. (2008). Mechanical and tribological properties of multi-element. Surf. Coat. Technol..

[68] Lai C.H., Lin S.J., Yeh J.W., Davison A. (2006). Effect of substrate bias on the structure and properties of multi-element (AlCrTaTiZr)N coatings. J. Phys. D Appl. Phys..

[69] Lin C.H., Duh J.G., Yeh J.W. (2007). Multi-component nitride coatings derived from Ti-Al-Cr-Si-V target in rf magnetron sputter. Surf. Coat. Technol..

[70] Chang H.W., Huang P.K., Yeh J.W., Davison A., Tsau C.H., Yang C.C. (2008). Influence of substrate bias, deposition temperature and post-deposition annealing on the structure and properties of multi-principal-component (AlCrMoSiTi)N coatings. Surf. Coat. Technol..

[71] Tsai M.H., Lai C.H., Yeh J.W., Gan J.Y. (2008). Effects of nitrogen flow ratio on the structure and properties of reactively sputtered (AlMoNbSiTaTiVZr)Nx coatings. J. Phys. D Appl. Phys..

[72] Tsai D.C., Chang Z.C., Kuo L.Y., Lin T.J., Lin T.N., Shieu F.S. (2013). Solid solution coating of (TiVCrZrHf)N with unusual structural evolution. Surf. Coat. Technol..

[73] Braic V., Vladescu A., Balaceanu M., Luculescu C.R., Braic M. (2012). Nanostructured multi-element (TiZrNbHfTa)N and (TiZrNbHfTa)C hard coatings. Surf. Coat. Technol..

[74] Huang P.K., Yeh J.W. (2010). Inhibition of grain coarsening up to 1000 °C in (AlCrNbSiTiV)N superhard coatings. Scr. Mater..

[75] Huang P.K., Yeh J.W. (2009). Effects of substrate bias on structure and mechanical properties of (AlCrNbSiTiV)N coatings. J. Phys. D Appl. Phys..

[76] Shen W.J., Tsai M.H., Chang Y.S., Yeh J.W. (2012). Effects of substrate bias on the structure and mechanical properties of (Al1.5CrNb0.5Si0.5Ti)Nx coatings. Thin Solid Films.

[77] Shen W.-J., Tsai M.-H., Yeh J.-W. (2015). Machining Performance of Sputter-Deposited (Al0.34Cr0.22Nb0.11Si0.11Ti0.22)50N50 High-Entropy Nitride Coatings. Coatings.

[78] Yeh J.-W., Lin S.-J. (2018). Breakthrough applications of high-entropy materials. J. Mater. Res..

